# Emulsion Stabilization with Functionalized Cellulose Nanoparticles Fabricated Using Deep Eutectic Solvents

**DOI:** 10.3390/molecules23112765

**Published:** 2018-10-25

**Authors:** Jonna Ojala, Miikka Visanko, Ossi Laitinen, Monika Österberg, Juho Antti Sirviö, Henrikki Liimatainen

**Affiliations:** 1 Fibre and Particle Engineering Research Unit, University of Oulu, P.O. Box 4300, FI-90014 Oulu, Finland; jonna.ojala@oulu.fi (J.O.); miikka.visanko@oulu.fi (M.V.); ossi.laitinen@oulu.fi (O.L.); juho.sirvio@oulu.fi (J.A.S.); 2 Department of Bioproducts and Biosystems, Aalto University, P.O. Box 16300, FI-00076 Aalto, Finland; monika.osterberg@aalto.fi

**Keywords:** cellulose nanocrystals (CNCs), cellulose nanofibrils (CNFs), nanoparticle, stabilization, *o*/*w* emulsion, surface-functionalization, deep eutectic solvent (DES)

## Abstract

In this experiment, the influence of the morphology and surface characteristics of cellulosic nanoparticles (i.e., cellulose nanocrystals [CNCs] and cellulose nanofibers [CNFs]) on oil-in-water (*o*/*w*) emulsion stabilization was studied using non-modified or functionalized nanoparticles obtained following deep eutectic solvent (DES) pre-treatments. The effect of the oil-to-water ratio (5, 10, and 20 wt.-% (weight percent) of oil), the type of nanoparticle, and the concentration of the particles (0.05–0.2 wt.-%) on the oil-droplet size (using laser diffractometry), *o*/*w* emulsion stability (via analytical centrifugation), and stabilization mechanisms (using field emission scanning electron microscopy with the model compound—i.e., polymerized styrene in water emulsions) were examined. All the cellulosic nanoparticles studied decreased the oil droplet size in emulsion (sizes varied from 22.5 µm to 8.9 µm, depending on the nanoparticle used). Efficient *o*/*w* emulsion stabilization against coalescence and an oil droplet-stabilizing web-like structure were obtained only, however, with surface-functionalized CNFs, which had a moderate hydrophilicity level. CNFs without surface functionalization did not prevent either the coalescence or the creaming of emulsions, probably due to the natural hydrophobicity of the nanoparticles and their instability in water. Moderately hydrophilic CNCs, on the other hand, distributed evenly and displayed good interaction with both dispersion phases. The rigid structure of CNCs meant, however, that voluminous web structures were not formed on the surface of oil droplets; they formed in flat, uniform layers instead. Consequently, emulsion stability was lower with CNCs, when compared with surface-functionalized CNFs. Tunable cellulose nanoparticles can be used in several applications such as in enhanced marine oil response.

## 1. Introduction

Emulsions are formed when two immiscible liquids are mixed and the droplets of one liquid are distributed in the continuous phase of another medium. In the case of oil-in-water (*o*/*w*) emulsions, oil droplets are dispersed in the aqueous phase [1]. Without stabilizers in emulsion, the dispersed oil droplets tend to coalesce gradually leading eventually to emulsion breaking [2,3,4,5,6]. Therefore, soluble surfactants, solid nano- or microsized particles or natural polymers (such as proteins or polysaccharides) are used for the thermodynamic and kinetic stabilization of *o*/*w* emulsions. Emulsions in general, and emulsion stabilization have widely been studied since last century, but the Pickering stabilization mechanism, where emulsion is kinetically stabilized with solid particles instead of soluble surfactants, gained more interest only a couple of decades ago as a result of advances in nanoparticle and material sciences [7,8,9]. Both inorganic (e.g., silica [4], titanium dioxide [10], different clays [11]) and organic nanoparticles (e.g., chitosan [6], cellulose [12], quinoa starch [13], curcumin [14] and cyclodextrins [15]) for example, have been reported to stabilize *o*/*w* emulsions by adsorbing irreversibly into the oil-water interface preventing coalescence more efficiently than traditional soluble surfactants [16]. Emulsions stabilized by various nano- or microscale particles, offer promising applications in fields that require high stability and low toxicity [17]. The use of nanoparticles as one tool to capture the enzymes or other catalysts to enhance the formation of Pickering emulsions and improve their catalytic activity have gained interest among researchers [18,19,20]. These techniques open new possibilities in applications where the synthesis of highly stable colloidosomes for example through dopamine polymerization can be done in the interfaces of Pickering emulsions [21].

Cellulose nanoparticles—including cellulose nanocrystals (CNCs) and cellulose nanofibers (CNFs) made from renewable cellulosic substrates—have been considered to be promising green and sustainable alternatives for *o*/*w* emulsion stabilization [22,23,24,25,26]. Even though, due to their inherent amphiphilic nature, cellulose nanoparticles can stabilize *o*/*w* emulsions without any surface chemical treatments [27], the topochemical surface modification of cellulosic nanoparticles can be implemented to further enhance the interaction of cellulose nanoparticles with highly hydrophobic matrixes such as mineral oils [28,29,30]. Previously, cellulose nanoparticles with hydrophobic surface functionalization have been reported to efficiently prevent the oil droplet coalescence in emulsion [5,31,32]. Surface modification e.g., hydrophobization typically improves nanoparticle wettability on an oil interface, which, in turn, promotes the formation of a physical hindrance between the oil drops. Actually, that is one of the key factors associated in Pickering stabilization; particles have to be properly wetted by both oil and water phases [33], but too high level of hydrophobicity will lead to water-in-oil (*w*/*o*) emulsion [16] or aggregation of particles [9] and, therefore, the hydrophobicity level has to be adjusted according to application. In addition, the stability of Pickering emulsions is attributed to the particle surface characteristics among their size, shape and origin. It is agreed that the particles which cover the oil droplets have to be small enough for effective Pickering stabilization to occur, and that by combining nanoparticles of different sizes and shapes, stabilization properties can be enhanced [12,34]. It has been suggested that larger particles will improve steric hindrance and prevent coalescence, whilst smaller particles will regulate the size of the oil droplets [12,23]. The emulsion stabilization depends also on particle concentration and particle-particle interactions as Horozov and Binks suggested [35]. Higher particle concentrations tend to form mechanical barriers and prevent droplet coalescence. Varanasi et al. studied the electrostatic stabilization with cellulose nanocrystals and they found out that the screening the surface charge affected the concentration of nanoparticles required for stable emulsion. They reported the reduction in electrostatic repulsion by salt addition which lead to permanent emulsion stabilization already at lower concentrations of CNCs [36]. Nanocelluloses, deriving from cellulose, have become one promising nanomaterial for various different applications due to their, unique characteristics and versatility [37,38]. Cellulose nanocrystals are typically produced by acid hydrolysis, and applications where they can be used are for example biomedical, wastewater treatment or in electronics [39,40]. Recent research have extended the use of CNCs in oil and gas industry, where cellulose-based nanoparticles can stabilize the hydrophobic oil molecules on water environment making the natural biodegradation of oil by marine microbes more efficient [32,41,42]. CNFs, that are produced through enzyme or other pre-treatment aided mechanical system (for example homogenizer, grinder or microfluidizator) possess unique characteristics, such as high aspect ratio and web-like structures, which enable its use in aerogels, films and membranes among other new applications like energy storage materials [43].

Despite of the applicability of deep eutectic solvents (DESs), only few studies can be found on cellulose nanoparticles produced with renewable chemicals such as DESs [44,45,46,47]. Cellulose nanoparticles produced in DESs in *o*/*w* emulsion stabilization applications have not yet been studied much [48]. However, studies can be found in DES functionalized nanoparticles, for example ammonium and phosphonium-based DES-functionalized graphene with enhanced electrical or mechanical properties [49] or carbon nanotubes functionalized in allyl triphenyl phosphonium bromide-based DES to better adsorb Hg^2+^ from water [50]. In addition to non-harmfulness, DESs are found to be non-flammable, biodegradable, possessing low toxicity and therefore providing one cost-effective route for nanoparticle production for *o*/*w* emulsion stabilization [51].

The aim of this research was to compare the *o*/*w* stabilization performance of cellulose nanoparticles (CNFs and CNCs) produced in DES system. Specifically, the stabilization mechanisms for nanoparticles with differences in morphology and hydrophobic/hydrophilic surface characteristics were addressed. The CNFs and CNCs utilized were fabricated from cellulose using three different deep eutectic solvent (DES) solutions as a pre-treatment and as a reaction medium for surface functionalization. The first sample was non-modified CNFs without any derivatization; the second one anionic, succinylated CNFs; the third one anionic, carboxylated CNCs. Commercially available CNCs from dissolving softwood pulp were used as a reference material to be compared with samples fabricated in DES. The morphology of the DES produced nanoparticles were studied using transmission electron microscopy (TEM). The stability against creaming of the nanoparticle-stabilized *o*/*w* emulsions were analyzed with a centrifugal analyzer. The oil droplet size and the size distribution were determined via a laser diffraction particle-size analyzer. In order to analyze the stabilization mechanism of the nanoparticles further, the nanoparticle morphology on the oil droplets was imaged using field emission scanning electron microscopy (FESEM), with polymerized styrene being utilized as a model compound.

## 2. Materials and Methods

### 2.1. Materials

Softwood dissolving pulp dry sheets (cellulose: 96.2%, hemicelluloses: 3.5%, total lignin: <0.5%, acetone soluble extractives: 0.17%; Domsjö Fabriker AB, Domsjö, Sweden) were disintegrated in deionized water, according to the ISO 5263-1:2004 standard. The disintegrated pulp was then filtered, washed with technical ethanol for 30 min, filtrated, and dried in an oven at 60 °C for 24 h. The reference CNCs (BGB ultra), produced using an oxidative process from acetate-grade dissolving pulp (Western hemlock), were purchased from Blue Goose Biorefineries Inc. (Saskatoon, SK Canada). Choline chloride (C_5_H_14_ClNO) (>98.0%) was obtained from TCI Europe (Eschborn, Germany), with urea (>99.0%), lithium chloride (LiCl) (99%), and oxalic acid dihydrate (C_2_H_2_O_4_∙2H_2_O) being sourced from Sigma Aldrich (Darmstadt, Germany), and uranyl acetate (98%) being obtained from Polysciences Europe GmbH (Hirschberg an der Bergstraβe, Germany). The ethanol (96%) for the washing procedures was bought from VWR (Helsinki, Finland), while succinic anhydride (>95%) was obtained from TCI Europe (Zwijndrecht, Belgium). Styrene (C_8_H_8_) (>99.0%) and benzoyl peroxide (C_14_H_10_O_4_) (>75%) were purchased from TCI Europe (Zwijndrecht, Belgium). All the chemicals were pro-analysis grade and were used without further purification. Deionized water was used throughout the experiments. Lightweight marine diesel oil was obtained from Neste Oyj (Kemi, Finland). The oil selected was a sulfur-free, winter-grade oil with a density of 828 kg/m^3^ at 15 °C, a viscosity of 1.846 mm^2^/s at 40 °C, and a conductivity of 0.073 µS/cm at 20 °C.

### 2.2. Fabrication of Cellulose Nanoparticles Using DES Pre-Treatments

**Figure 1:**
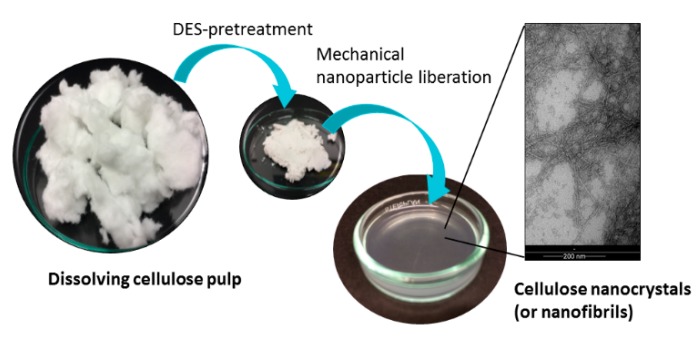
The schematic description of the fabrication stages of nanocellulose samples.

The nanocellulose samples were fabricated through different DES pre-treatments. In Figure 1, the schematic fabrication route is presented. Briefly, the ethanol washed and oven-dried dissolving pulp was immersed into DES and after reaction, the obtained sample was further diluted with water in desired consistency and the individual cellulose nanocrystals or cellulose nanofibers were liberated mechanically.

#### 2.2.1. Chemically Unmodified CNFs

**Figure 2:**
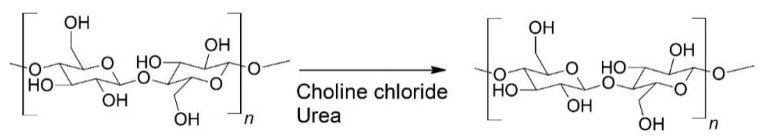
Non-derivative pre-treatment of cellulose with urea-choline chloride DES.

Non-derivatizing urea-choline chloride pre-treatment [44] was used for the preparation of the CNFs without surface functional groups. DES based on urea-choline chloride was produced by heating 64.8 g choline chloride and 48.9 g urea in a round-bottom flask at 100 °C in an oil bath for about 15 min in order to obtain a clear, colorless liquid. The cellulose sample (1.13 g of dry sheets of pulp) was added to the liquid and treated at 100 °C for 2 h (Figure 2).

After the DES treatment, 200 mL of water was added while mixing, and the pulp was filtered (Whatman 413) and washed with deionized water (2000 mL). The sample was further disintegrated into individual nanofibers using a microfluidizer (M-110EH-30, Microfluidics, Westwood, MA, USA). The sample was diluted in a low concentration (0.1%) before disintegration to avoid clogging of the chambers, and it was then passed through 400 µm + 200 µm chambers three times at a pressure of 1000 bar, before being passed through a combination of 400 µm and 100 µm chambers twice at a pressure of 1600 bar. This dilute, yet blurry, non-functionalized CNF suspension was labeled as NON-F-CNF.

#### 2.2.2. Surface Functionalized CNFs

**Figure 3:**
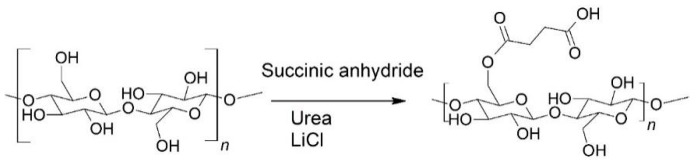
Succinylation reaction of cellulose in urea-LiCl DES.

Succinylation in DES of urea-LiCl was applied as a pre-treatment for the fabrication of surface functionalized CNFs [46]. The urea-LiCl DES was prepared by mixing 131.45 g urea and 18.55 g LiCl (5:1 molar ratio), and heating the mixture at 80 °C in an oil bath until a clear, colorless liquid was formed. The temperature of the DES solution was decreased to 70 °C prior to the reaction. The dissolving pulp (1.50 g) and succinic anhydride (9.27 g) (at a molar ratio of 1:10) were mixed into the solution under continuous stirring for 6 h (Figure 3). Following the reaction, the mixture was removed from the oil bath and 150 mL ethanol was added. The resulting mixture was filtered, and the modified pulp was washed with ethanol (100 mL) and deionized water (1500 mL). The pH was adjusted to 8 (originally 5.2), with 0.25 M NaOH, and the pulp was passed through the Microfluidizer three times at 1 wt.-% concentration, with chamber combinations of 400 µm + 200 µm and 400 µm + 100 µm, and pressures of 1000 bar and 1600 bar, respectively. The anionic, surface-functionalized CNF sample as achieved was a thick, gel-like solution, and was labeled as SF-CNF.

#### 2.2.3. Surface Functionalized CNCs

**Figure 4:**
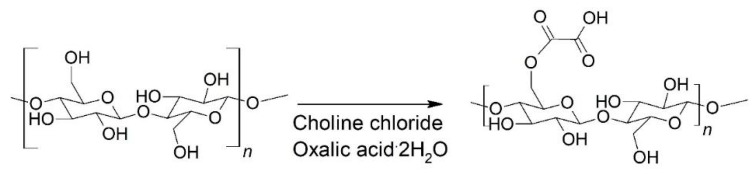
The carboxylation reaction of cellulose in choline chloride-oxalic acid (dihydrate) DES.

The CNCs were produced using a hydrolytic DES pre-treatment based on oxalic acid and choline chloride [45]. The DES was produced by mixing choline chloride (63.06 g) and oxalic acid dihydrate (56.94 g), and heating the mixture at 100 °C for approximately 30 min until a clear solution was formed. Dissolving pulp (oven-dry weight 1.2 g) was added, and the mixture was heated for 6 h (Figure 4). The suspension was then cooled and 100 mL of water was added. The sample was filtered and washed with 1000 mL of water. The sample was diluted to 1 wt.-% concentration, and the pH was adjusted to 7, with 0.25 M NaOH. The sample was passed through 400 µm + 200 µm chambers at 1000 bar twice, before being passed through 400 µm + 100 µm chambers twice at 1500 bar and through 200 µm + 87 µm chambers once at 1700 bar. Following mechanical disintegration, the sample appeared slightly viscous and almost transparent, and was labeled as SF-CNC. All the cellulose nanoparticle specimens used in the experiment are summarized in Table 1 in the results section.

### 2.3. Determination of Acidic Groups and Calculation of Nanoparticle Yields

The carboxyl content of the ethanol-washed raw pulp and the DES-pre-treated dissolving cellulose samples was determined via the conductometric titration protocol described by Katz et al. and Rattaz et al. [52,53]. The yield of each nanoparticle synthesis were calculated comparing the dry mass of the final product to the original dry mass of the cellulose.

### 2.4. Transmission Electron Microscopy (TEM)

The morphological characteristics of the fabricated nanoparticles were analyzed with a Tecnai G2 Spirit transmission electron microscope (FEI Europe, Eindhoven, The Netherlands). The samples were prepared by first diluting each cellulose nanoparticle suspension with deionized water. A 7 µL droplet of 0.01 M polylysine suspension was added to the carbon-coated copper grid and after that a 7 µL droplet of diluted nanoparticle suspension was dosed on the copper grid and any excess sample was removed by touching the droplet gently with the corner of a filter paper. The samples were negatively stained with a droplet of uranyl acetate (2% *w*/*v*) placed on top of each sample and removing the excess uranyl acetate with a filter paper. After drying the grids under room temperature, they were analyzed at 100 kV under standard conditions. Images were captured with a Quemesa CCD camera and iTEM image analysis software (CE, Olympus Soft Imaging Solutions GmBH, Munster, Germany) was used to measure the average widths and lengths of the nanoparticles. The final results were averaged and standard deviations were calculated.

### 2.5. Diffuse Reflectance Infrared Fourier Transform (DRIFT) Spectroscopy

Chemical characterization of the dissolving cellulose and DES-pre-treated celluloses (before the mechanical liberation of the nanoparticles) was performed using DRIFT spectroscopy. The spectra were collected from freeze-dried samples with a Bruker Vertex 80v spectrometer (Billerica, MA, USA) in the 600–4000 cm^−1^ range. Forty scans were taken for each sample at a resolution of 2 cm^−1^.

### 2.6. Water Contact Angles of Self-Standing Cellulose Nanoparticle Films

Static sessile-drop contact-angle measurement was used to determine the hydrophilicity levels of the fabricated nanoparticles. Self-standing films were prepared from nanoparticle suspensions through dispersion with an Ultra-Turrax mixer at 10,000 rpm for 3 min. The suspensions were vacuum filtrated in a glass filter funnel (diameter: 7.2 cm) using a filter membrane (polyvinylidene fluoride, 0.65 μm, Millipore Durapore, Alsace, France). The films were dried overnight at room temperature. The contact angles were achieved by using Milli-Q water as a probe liquid.

During the measurement process, a CAM 2000 (KSV Instruments Ltd., Helsinki, Finland) system equipped with a high-speed CCD video camera and analysis software was used. The water contact angle (WCA) was determined as a function of time by dropping a water droplet (volume: 6.5 µL) on the film surface. The WCA was measured for 60 s, but due to possible bending of the nanoparticle films when wetted, the contact angle at 5 s was used for comparison. The Laplace/Young method was used in calculations [54]. For each sample, four droplets were measured in different locations and the standard deviation was then calculated from the averaged results.

### 2.7. Surface Tension by Tensiometer

The changes in the surface tension of aqueous nanoparticle suspensions as a function of concentration at room temperature was measured using The du Noüy ring method [55]. A platinum wire ring was submerged into the nanocellulose suspension and the net fluid force on the ring during withdraw was measured. The force (*F*) that was required to raise the ring from the liquid was measured and related to the liquid’s surface tension (*γ*), in accordance with Equation (1):

(1) $$F=2\pi \cdot \left(r_{i}+r_{o}\right)\cdot \gamma$$

where *r_i_* is the radius of the inner ring of the liquid film pulled and *r_o_* is the radius of the outer ring of the liquid film.

### 2.8. Fabrication of Nanoparticle-Stabilized o/w Emulsions

Cellulose nanoparticle solutions were diluted with deionized water in order to obtain target concentrations of 0.05–0.2 wt.-%. Oil was then added at fixed concentrations of 5%, 10%, and 20% (wt.-%), and emulsified using an UltraTurrax mixer at 7000 rpm for 15 min.

### 2.9. Properties of the Stabilized o/w Emulsions

Emulsion stability was evaluated by measuring creaming and coalescence phenomena within *o*/*w* emulsions using an analytical centrifuge (LUMiFuge, L.U.M. GmbH, Berlin, Germany) at 20 °C. A rotational speed of 1200 rpm was used, corresponding to a centrifugal force of 205 G. During centrifugation, a near-infrared sensor measures light transmission at a wavelength of 800 nm through horizontally positioned sample cells. Changes in transmission over the whole cell length are used to indicate the separation process as a function of time. Two parallel measurements were analyzed for each sample.

The size distribution of the oil droplets in the stabilized *o*/*w* emulsions was measured with a laser diffraction particle size analyzer (LS 13 320, Beckman Coulter, Brea, CA, USA). Each sample was measured directly after dispersing the oil into water, with stabilizing nanoparticles present in the solution. The mean droplet diameter was taken from three replicate measurements.

Styrene/water emulsions were prepared using SF-CNFs and REF-CNCs in order to investigate the stabilizing mechanisms of different nanoparticles. To enable imaging of the nanoparticle-stabilized emulsions, styrene was polymerized via a reaction with benzoyl peroxide, following a similar methodology to that reported by Alduncin et al. [56]. In brief, 4.78 g of styrene, 0.12 g of benzoyl peroxide, cellulose nanoparticles (oven-dry weight 0.04 g) equivalent to 0.2% dosage and deionized water were added to achieve a total weight of 20 g. The emulsions were produced by sonicating the suspension for 2 min and polymerization was carried out under magnetic stirring at a constant temperature of 75 °C. Dilutions were made using the polymerized solutions and deionized water, and the remaining styrene was allowed to evaporate. From the dilutions, small droplets were sampled for FESEM imaging (ULTRA plus, Zeiss, Oberkochen, Germany).

## 3. Results and Discussion

### 3.1. Characterization of Cellulose Nanoparticles

The cellulose nanoparticles used in the present study were cellulose nanofibers (NON-F-CNF and SF-CNF) and cellulose nanocrystals (SF-CNC and REF-CNC) with variable charge contents (i.e., both the morphology and charge properties of the nanoparticles were varied). The carboxylic-acid content of the DES-pre-treated specimens varied from 0.17–0.67 mmol g^−1^ (Table 1). The REF-CNCs had a previously determined carboxylic-acid content of 0.15 mmol g^−1^—close to that of the SF-CNCs [48]. The succinylated SF-CNF was the only nanoparticle specimen with a notably higher charge density (CD). Reaction yields after the DES pre-treatments varied from 75–99%, depending on the DES system. There was a lower yield for the SF-CNCs when compared with the other specimens due to the dissolution of amorphous regions. Despite this, the yield for the SF-CNCs in this study was higher than that typically reported for the mineral acid hydrolysis of nanocrystals [57,58].

**Figure 5:**
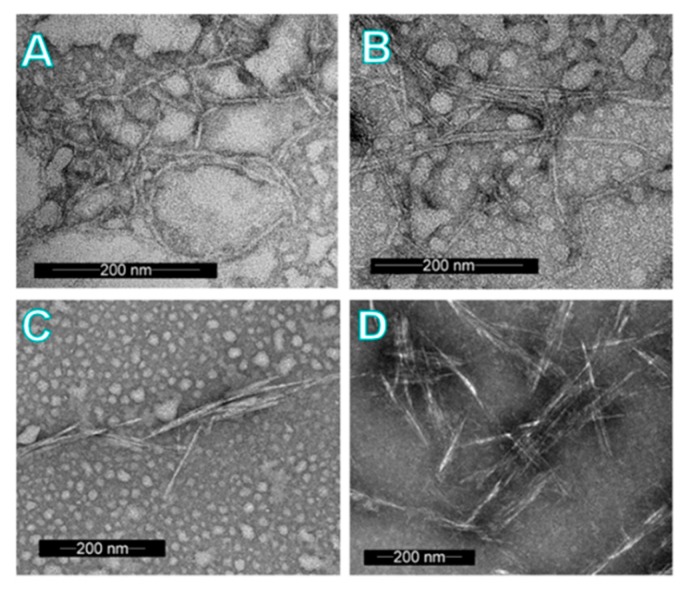
TEM images of the cellulose nanoparticles (**A**–**D**). Upper figures are representing the CNF samples NON-F-CNF (**A**) and SF-CNF (**B**) while lower figures represent the samples SF-CNC (**C**) and REF-CNC (**D**).

Based on the TEM images (Figure 5) of the nanoparticles, the NON-F-CNFs and SF-CNFs appeared to be elongated and flexible nanofibrous structures, whereas the SF-CNCs and REF-CNCs consisted of shorter and stiffer individual nanocrystals, which were rod-like. The NON-F-CNFs and SF-CNCs had similar widths of around 3–5 nm, while the SF-CNF nanofibers had even smaller average diameters of 2–3 nm. The widths of the REF-CNCs varied from 3–8 nm, with the lengths being around 50–350 nm—similar to those of SF-CNCs [48].

**Figure 6:**
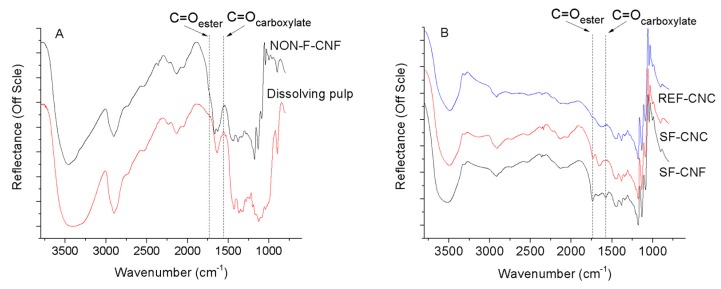
DRIFT spectra for pure dissolving cellulose pulp and non-functionalized CNF (**A**) and cellulosic nanoparticles REF-CNC, SF-CNC and SF-CNF (**B**). The most distinct bands are numbered and presented as dashed lines: 1730 cm^−1^ is associated with C=O stretching in the carboxylic acid and ester groups, and 1574 cm^−1^ is associated with the C=O stretching of carboxylate anions.

The DRIFT spectra for the dissolving pulp, the REF-CNCs and the DES-pre-treated samples are presented in Figure 6. All of the spectra for the cellulose samples were typical, with characteristic OH stretching bands appearing at 4000–2995 cm^−1^ and CH stretching bands appearing at 2900 cm^−1^ (Figure 2A). In addition, successful functionalization was seen with SF-CNFs and SF-CNCs (Figure 2B), with bands at 1730 cm^−1^ (associated with C=O stretching in the carboxylic acid and ester groups) and 1574 cm^−1^ (associated with the C=O stretching of carboxylate anions).

### 3.2. Wetting Characteristics of Cellulose Nanoparticle Films

**Figure 7:**
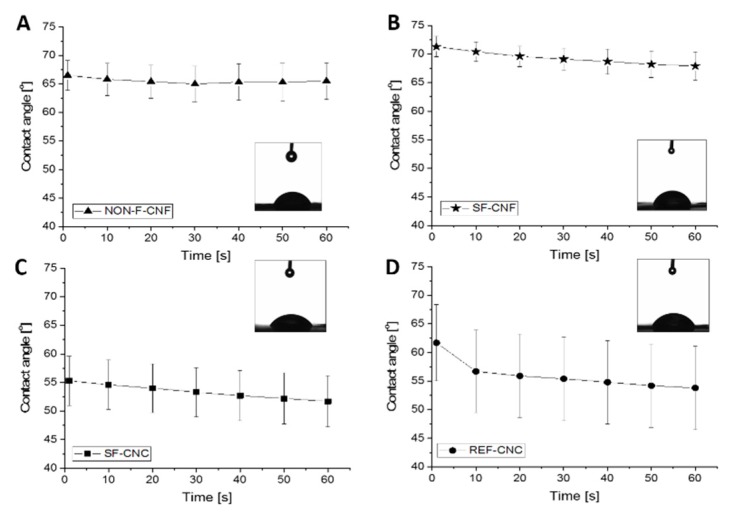
Time dependence of the water contact angles (WCAs) measured on the (**A**–**C**) DES-pre-treated nanoparticle film surfaces and (**D**) reference CNC film surface. The physical shapes of the water droplets at t = 5 s are provided.

The hydrophilicity levels (i.e., the wetting characteristics) of the cellulose nanoparticles were evaluated by measuring the time dependent WCAs on the cellulose nanoparticle films (Figure 7). Slight decrease with time of the WCAs on nanocellulose surfaces was detected during 60 s measuring time. There may occur some membrane bending due to wetting in long measurements and for that reason, the contact angles at 5 s were used for comparison. The highest WCA was measured with SF-CNF being 71°. At the same time, other samples NON-F-CNF, SF-CNC and REF-CNC had the contact angles of 66°, 55° and 57°, respectively. SF-CNF was the least hydrophilic sample while having the highest surface charge. Overall, the contact angles were higher with these DES pre-treated nanocelluloses compared to that made with oxidizing methods. It is reported, e.g., dicarboxylic acid cellulose-nanofibril membranes have had an average water contact angle of 45.0°, and crystalline nanocellulose membranes 27–44° [59,60]. However, according to previous studies, nanoparticles with WCAs of 40–60° are considered optimal for the stabilization *o*/*w* emulsions, as the stabilizing agent is able to interact efficiently with both phases in the dispersion system [27].

### 3.3. Surface Tension of Nanoparticle Solutions

**Figure 8:**
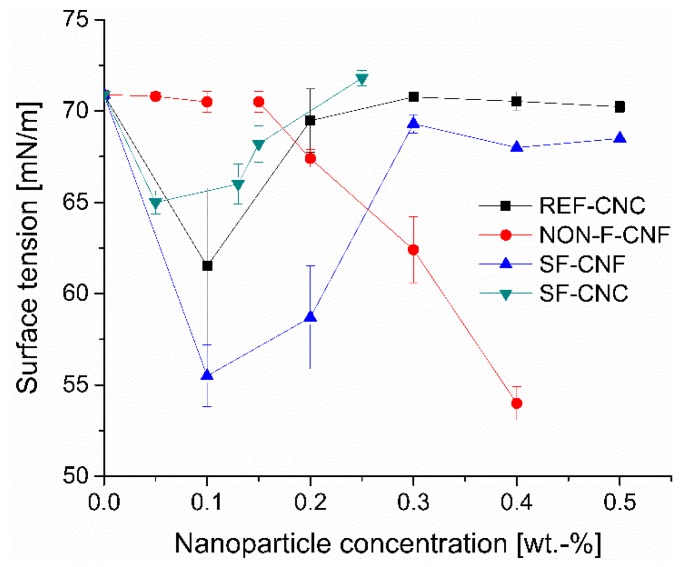
The surface tension of water with increasing nanoparticle concentrations.

The surface activity of nanoparticles was determined by measuring the surface tension of the aqueous cellulose nanoparticle suspensions at different concentrations (0.05–0.5 wt.-%) via the du Noüy ring method (Figure 8). All the nanoparticles affected the surface tension of the water solution, but the surface-tension response to the increase in nanoparticle concentration varied. The surface tension for the NON-F-CNFs remained constant at low concentrations but started to decrease linearly with concentrations of 0.2 wt.-% and above. Ultimately, a reduction of almost 24% was achieved at 0.4 wt.-% nanoparticle concentration.

The SF-CNFs, SF-CNCs, and REF-CNCs, all of which possessed same or higher hydrophilicity levels, behaved differently from the NON-F-CNFs. Decreases of 22%, 8%, and 12%, respectively, were observed at low concentrations (surface tension minimum was reached already at nanoparticle concentrations of 0.05 wt.-%, 0.10 wt.-%, and 0.10 wt.-%, respectively). Further increases in the nanoparticle dosages changed the surface tension so that it approached the level of pure water. This phenomenon can be explained by the accumulation and aggregation of functionalized nanoparticles at the air-liquid interface, a finding that has also been reported in relation to other nanoparticles [61,62,63]. Moreover, it has been reported that larger particles may have a more marked effect on the surface tension than smaller ones at the same concentration as seen in this case with SF-CNFs vs. SF-CNCs and REF-CNCs [16,62,63]. The different behavior of NON-F-CNFs may originate in its stronger inter-particle interactions—i.e., it is produced gradually in an organized monolayer because of its lower hydrophilicity, while the other nanoparticles form in less uniform structures or aggregates at higher concentrations.

### 3.4. Properties of Oil Droplets in Nanoparticle-Stabilized Emulsions

Typically, an *o*/*w* emulsion without a nanoparticle stabilizer or surfactant will coalesce quickly because there is no hindrance from the oil/water interface that could prevent the separate phases from coming into contact. Cellulose nanoparticles are distributed on the oil-droplet surface, thus increasing steric and/or charge hindrance, and stabilizing the formed droplets at certain sizes. In the present study, the *o*/*w* emulsions were formed using cellulose nanoparticles as stabilizers at fixed concentrations of 0.05 wt.-%, 0.1 wt.-%, and 0.2 wt.-%, and three oil concentrations of between 5 wt.-% and 20 wt.-%. Notably, the oil-droplet size was affected by the cellulose nanoparticle type and concentration. The largest oil-droplet sizes (i.e., those with less-efficient emulsification effects) were recorded when REF-CNCs and NON-F-CNFs were used (Table 2). Additionally, an increase in the nanoparticle dose from 0.05 wt.-% to 0.2 wt.-% decreased the oil-droplet size more clearly with emulsions stabilized with both CNCs and SF-CNF samples, when compared with the NON-F-CNF. The smallest droplet sizes were obtained using functionalized SF-CNFs at a concentration of 0.2 wt.-%. When keeping the nanoparticle dosage constant, the oil-droplet size was noted to increase gradually as more oil was added to the emulsion. When the oil water ratio was kept similar, the oil droplets were smaller if more nanoparticles were present. According to this trend, it can be assumed that larger amount of nanoparticles is needed to stabilize the oil droplets to smaller size i.e. the nanoparticle concentration is restricting the oil droplet size. Some exceptions might occur due to sampling issues or other problems.

The effects of nanoparticle concentrations and oil water ratio on oil droplet size distributions in stabilized emulsions are presented in the Supplementary data (Figure S1). When the concentration of nanoparticles increased from 0.05 wt.-% to 0.2 wt.-%, the droplets in smaller size range increased with SF-CNF and SF-CNC. NON-F-CNF, on the other hand performed similarly than SF-CNF and SF-CNC at the nanoparticle concentration 0.05 wt.-% but at 0.2 wt.-%, the size distribution widened and the highest volumes were found at larger droplets’ size range. REF-CNC had a narrow distribution at 0.05 wt.-% with high volumes of larger (around 20–30 µm) droplets while at 0.2 wt.-%, the distribution of REF-CNC stabilized droplets was much wider. Even though there were changes in droplet sizes and distributions in emulsion, the differences were not that clear for conclusions to be drawn. SF-CNF was performing well at both nanoparticle concentrations and even with the highest oil content, giving narrow distributions and small droplet sizes.

### 3.5. Stability of o/w Emulsions

**Figure 9:**
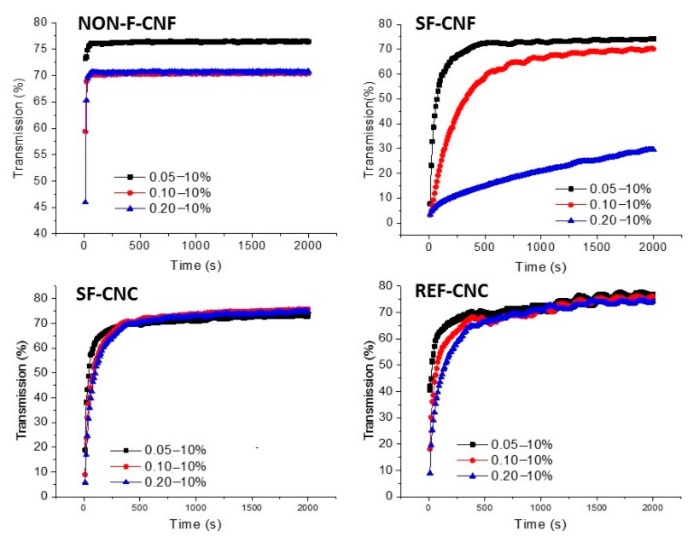
The stability of *o*/*w* emulsions against creaming as a function of centrifugation (205 G) time. Nanoparticle samples (NON-F-CNF, SF-CNF, SF-CNC and REF-CNC) at concentrations of 0.05 wt.-%, 0.1 wt.-%, and 0.2 wt.-% with a constant oil dosage of 10 wt.-% were used to stabilize the oil droplets.

Density-driven creaming is known to be the most typical route for phase separation in nanoparticle-stabilized *o*/*w* emulsions. Coalescence, on the other hand, is a de-emulsification process that occurs easily without a stabilizer in the emulsion. All the *o*/*w* emulsions stabilized by cellulose nanoparticles were stable (i.e., resistant to coalescence), as observed visually, but had a high tendency for creaming; as such, all the measurements were initiated immediately after emulsification. The lower transmissions at the end of the stability measurements with the analytical centrifuge indicate better stability in the emulsions (Figure 9). Moreover, the kinetics shown in the curve are also important; curves with a smaller slope present samples that resist creaming for a longer time.

In all the samples, a clear, aqueous phase started to form below the emulsified oil droplets during the first 30 min after emulsification. Creaming was fastest with the NON-F-CNFs, with the phenomenon occurring immediately after the stability analysis started. The SF-CNFs, on the other hand, slowed down the creaming, especially with the highest cellulose-nanoparticle concentration (0.2%) and 10 wt.-% oil. The differences between the stability kinetics became clearer as centrifugation progressed as shown in the Figure 9.

The CNC samples were not able to slow down the creaming as efficiently as the SF-CNFs, but they still performed better than the NON-F-CNFs. Moderate hydrophilicity and nanofibrous morphology seem, therefore, to improve the performance of cellulose nanoparticles as dispersing agents (stability performance: SF-CNFs > SF-CNCs ≥ REF-CNCs > NON-F-CNFs). These observations are in line with previously reported findings regarding functionalized CNFs with high anionic charges [64]. It has been found that CNCs prevent creaming more than emulsions stabilized with non-functionalized CNFs, which are, in fact, prone to creaming [26]. In addition, it has been suggested that thinner nanofibers (i.e., SF-CNFs vs. NON-F-CNFs) form denser webs around oil droplets, thereby providing a stronger mechanical block against coalescence [64].

The SF-CNFs displayed the best resistance to creaming. For example, the final transmittance decreased from approximately 70% to 30% at 10 wt.-% oil when the SF-CNF dose was increased from 0.05 to 0.20 wt.-% (Figure 9). This phenomenon did not occur with NON-F-CNFs, SF-CNCs, and REF-CNCs (final transmittance approximately 70–80% at all the dosages) but similar stability kinetics were observed with the other nanoparticles at all the dosages (although they all affected the oil-droplet size similarly to the SF-CNFs, Table 2). In addition, an increase from 0.05 up to 0.2 wt.-% in the SF-CNF concentration slowed down creaming at all studied oil concentrations (results not shown here.).

Visual observation of the *o*/*w* emulsions after stability testing revealed that the REF-CNCs were insufficient to prevent oil coalescence. Here, the oil formed a separate phase (Supplementary Materials, Figure S2). Similar de-stabilization was observed with NON-F-CNFs at all oil and nanoparticle concentrations (results not shown). In contrast, the SF-CNFs prevented coalescence completely at all of the oil dosages studied (i.e., 5, 10, and 20 wt.-%—Figure S2). The SF-CNCs prevented coalescence at low nanoparticle and oil concentrations of 0.05 wt.-% and 5 wt.-%, but coalescence did occur with higher doses. A similar trend was observed with a 0.1 wt.-% SF-CNC concentration, but only the 20 wt.-% oil dosage coalesced here. It was also found that, within optimal nanoparticle-to-oil ratio, the use of SF-CNCs resulted in better emulsion stability than the use of REF-CNCs. Based on visual observations, stabilized emulsion layer thickness varied between CNCs and CNFs, when the emulsion was centrifuged in similar conditions. It is assumed that CNFs form web-like structures that prevent droplets to pack so tightly than those stabilized with CNCs and therefore thicker layer of emulsion was formed with CNFs. For this reason, CNFs might provide higher stability towards temperature rise and slow down the creaming effect as well. Similar observations are reported also by Gestranius et al. (2017) [65].

### 3.6. Stabilization Mechanisms of Cellulose Nanoparticles

**Figure 10:**
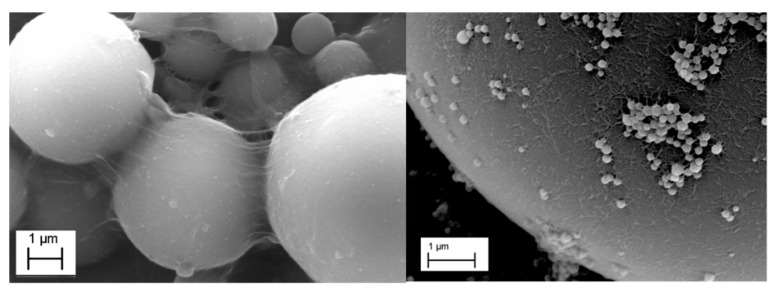
Polymerized styrene beads stabilized with SF-CNFs (**Left**) and REF-CNCs (**Right**) using a 0.2 wt.-% nanoparticle concentration.

Cellulose nanoparticles (SF-CNF and REF-CNC) were studied with FESEM to illustrate the surface morphology and the settling of nanoparticles to stabilize droplets in emulsion. Due to its high volatility, diesel oil was replaced here with styrene because it can be used as a model compound to simulate oil-droplet surface coverage in emulsion after a polymerization reaction [66]. Emulsion polymerization of styrene resulted in the creation of beads with a broad size distribution. The smallest beads were a few hundred nanometers, while the largest ones imaged were approximately 50 µm in diameter. The nanoparticles were clearly visible on the surface of the styrene beads (Figure 10). The REF-CNCs formed a homogeneous layer, whereas the SF-CNFs stabilized the beads by forming a web-like structure that was more heterogeneous and protruded out from the surface of beads. The layers of SF-CNFs also attached the beads together via a network of mechanically entangled nanofibers. It was also detected, that smaller CNCs stabilized more very small styrene beads that were attached in the surface of the larger beads, which is in line with previous findings. It has been suggested that the stable emulsion obtained by using nanoparticle stabilizers is the result of the combination of particles of different sizes [12]. The large particles are more responsible for preventing coalescence, while the smaller particles define the size of the oil droplets.

In these experiments, efficient *o*/*w* emulsion stabilization and stabilizing web structures were obtained only with nanofibrous SF-CNFs—the nanoparticles which had lower hydrophilicity. The NON-F-CNFs prevented neither coalescence, nor creaming, probably because of their low hydrophilicity level together with lack of sufficient surface charge. The CNC samples, on the other hand, had good interaction with both dispersion phases (due to their moderate hydrophilicity). The rigid structures of the CNCs, however, meant that voluminous web structures were not formed; rather, they led to flat, uniform layers being created on the surface of the oil droplets that have been noticed also in the work of Cherhal et al. [67]. Consequently, the stability levels for the CNC emulsions were lower when compared with surface-functionalized CNFs. The key findings of this study are presented in Table 3. All the cellulosic nanoparticles studied decreased the oil-droplet size (compared to oil in water emulsion without the stabilizer as measured earlir by Ojala et al. [32]) The decrease level was depending on the nanoparticle used.

## 4. Conclusions

In this study, we have reported the emulsion stabilization capacity and mechanisms of CNFs and CNCs fabricated and functionalized in DES for mostly irreversible particle stabilization technique. All the cellulose-nanoparticle stabilized emulsions formed creaming layers, but the creaming kinetics differed, depending on the type on nanoparticle, as the stability analysis showed. The functionalized SF-CNFs were found to be the most efficient in preventing creaming. In addition, the oil droplets were stable against coalescence with SF-CNFs only. The conventional chemicals used for functionalization are strong inorganic acids and organic solvents possessing high volatility. Because of the high risk associated to the use of such chemicals, it is important to develop safer and eco-friendlier alternatives which DESs definitely are. DESs provide one possible novel reaction media and functionalization route for cellulosic nanoparticles. The results showed the changes in surface chemistry after DES treatment. Replacing the traditional chemicals used in nanoparticle functionalization with non-toxic and cheap DESs open highly ecological as well as economically competitive possibilities.

In this research, it seemed that functionalization among the fibrillary forms of nanoparticles were the main features affecting oil-droplet stability and resistance to coalescence. There are still problems to solve, however, as the exact features of functionalized nanoparticles in oil stabilization remain unknown. In this regard, this paper provides a starting point for further investigation into the mechanisms of interaction in *o*/*w* emulsion with different types of nanoparticles produced in DES-system.

## Figures and Tables

**Table 1:** Cellulose nanoparticles production with DES pre-treatment, particle morphology, surface functionality, yield after pre-treatment, and carboxyl content.

Sample Label	Morphology	DES Treatment	Chemical Reaction	Yield [%]	Anionic Charge [mmol·g^−1^]
NON-F-CNF	Fibril	C_5_H_14_ClNO/Urea	No surface funct.	>99	0.28
SF-CNF	Fibril	Urea/LiCl + succinic anhydride	Succinylation	93	0.67
SF-CNC	Crystal	C_2_H_2_O_4_∙2 H_2_O/C_5_H_14_ClNO	Carboxylation	75	0.17
REF-CNC	Crystal	-	-	-	0.15

**Table 2:** Summary of the mean oil-droplet diameters (µm) for oil-to-water emulsions with varying nanoparticle concentrations and oil water ratio.

		Mean Droplet Diameter [µm]			
Nanoparticle Dosage [wt.-%]	Oil [wt.-%]	NON-F-CNF	SF-CNF	SF-CNC	REF-CNC
0.05	5	12 ± 4	12 ± 3	16 ± 4	22 ± 5
0.05	10	15 ± 4	15 ± 4	17 ± 4	26 ± 6
0.05	20	21 ± 4	15 ± 4	22 ± 5	27 ± 6
0.1	5	24 ± 6	10 ± 2	14 ± 4	21 ± 5
0.1	10	28 ± 7	11 ± 3	16 ± 4	20 ± 5
0.1	20	28 ± 7	14 ± 3	19 ± 5	25 ± 6
0.2	5	13 ± 5	9 ± 2	9 ± 3	17 ± 5
0.2	10	24 ± 6	10 ± 3	19 ± 5	19 ± 5
0.2	20	22 ± 6	11 ± 3	14 ± 4	22 ± 6

**Table 3:** CNC and CNF sample characteristics and performance in emulsion stabilization. The scale starts at the lowest/poorest performance (+) and moves up to very good performance (++++).

Sample	Structure	Hydrophilicity Level	Oil Droplet Size in Emulsion	Emulsion Stability against Coalescence	Stabilization Mechanism
NON-F-CNF	Fibrils	Low	+	not stable	-
SF-CNF	Fibrils	Very Low	++++	+++	web-formation
SF-CNC	Crystals	Moderate	+++	++	-
REF-CNC	Crystals	Moderate	++	+	Flat, homogeneous cover on oil surface

## Supplementary Materials

The following are available online; Figure S1: Particle size distributions of nanoparticle stabilized emulsions in two concentrations of nanoparticles (0.05 and 0.2 wt.-%) and three oil to water ratios 5, 10 and 20 wt.-%, Figure S2: *o*/*w* emulsions after the centrifugation (figure on the left) SF-CNF stabilized emulsions, starting from the: nanoparticle dosage of 0.05, 0.1 and 0.2 wt.-% (oil amount 10 wt.-%), and (figure on the right) REF-CNC stabilized emulsions, starting from the left: nanoparticle dosage of 0.05, 0.1 and 0.2 wt.-% (oil amount 10 wt.-%). The red colored liquid is the oil.
