# Supplementary material for: Emulsion Stabilization with Functionalized Cellulose Nanoparticles Fabricated Using Deep Eutectic Solvents
Source: Molecules. 2018 Oct 25;23(11):2765. doi: 10.3390/molecules23112765 (PMC6278293; doi:10.3390/molecules23112765)
Supplement: Supplementary file 1 [file molecules-23-02765-s001.pdf]

Supplementary data

Emulsion stabilization with functionalized cellulose nanoparticles fabricated using deep eutectic solvents

Jonna Ojala*, Miikka Visanko, Ossi Laitinen, Monika Österberg, Juho Antti Sirviö, and Henrikki Liimatainen

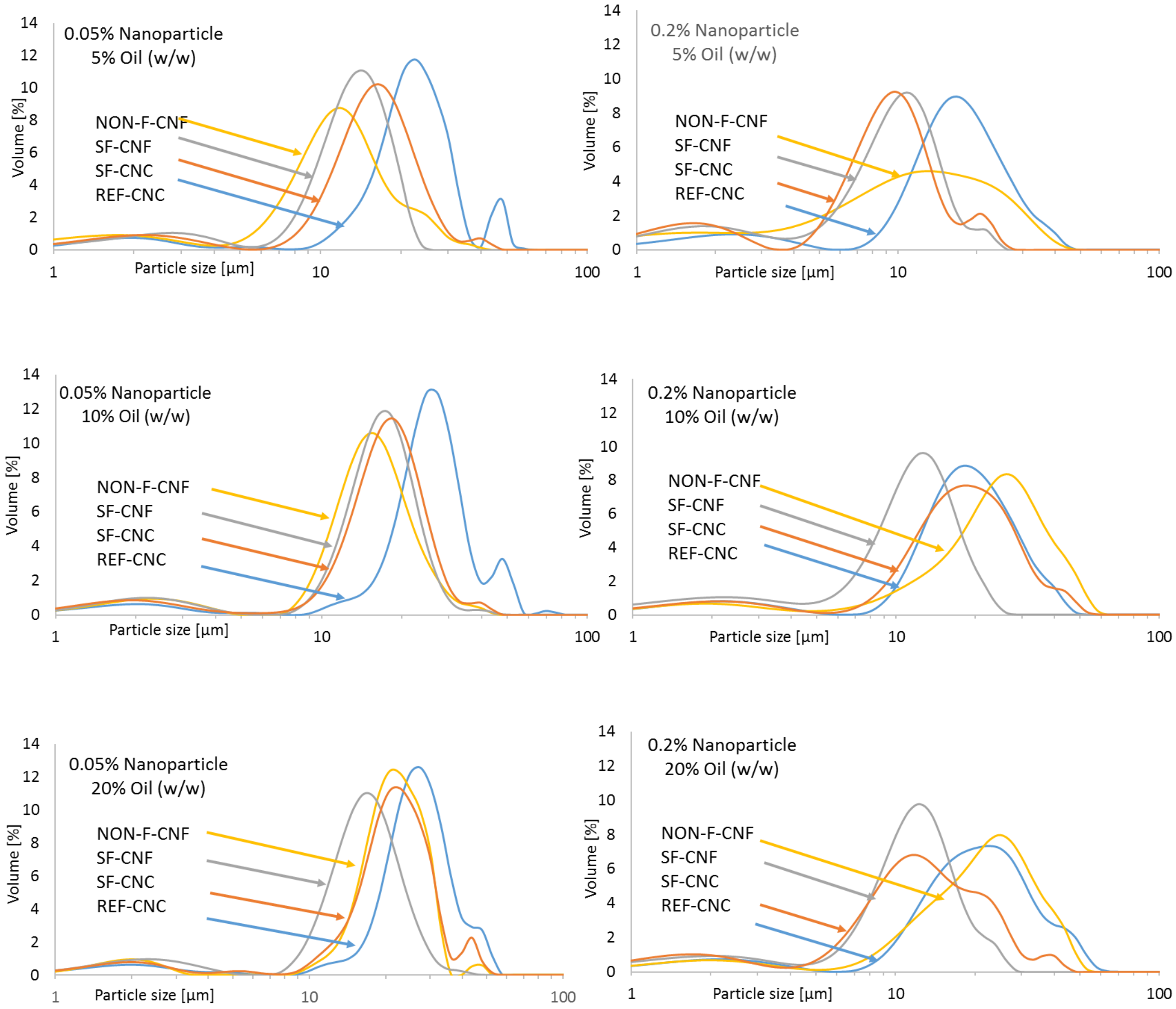

Figure S1: Particle size distributions of nanoparticle stabilized emulsions in two concentrations of nanoparticles (0.05% and 0.2% (w/w)) and three oil to water ratios 5%, 10% and 20% (w/w).

"The effects of nanoparticle concentrations and oil water ratio on oil droplet size distributions in stabilized emulsions are presented in the Supplementary data (Fig. S1). When the concentration of nanoparticles increased from 0.05% to 0.2%, the droplets in smaller size range increased with SF-CNF and SF-CNC. NON-F-CNF, on the other hand performed similarly than SF-CNF and SF-CNC at the nanoparticle concentration 0.05% but at 0.2%, the size distribution widened and the highest volumes were found at larger droplets´ size range. REF-CNC had a narrow distribution at 0.05% with high volumes of larger (around 20–30µm) droplets while at 0.2%, the distribution of REF-CNC stabilized droplets was

much wider. Even though there were changes in droplet sizes and distributions in emulsion, the differences were not that clear for conclusions to be drawn. SF-CNF was performing well at both nanoparticle concentrations and even with the highest oil content, giving narrow distributions and small droplet sizes."

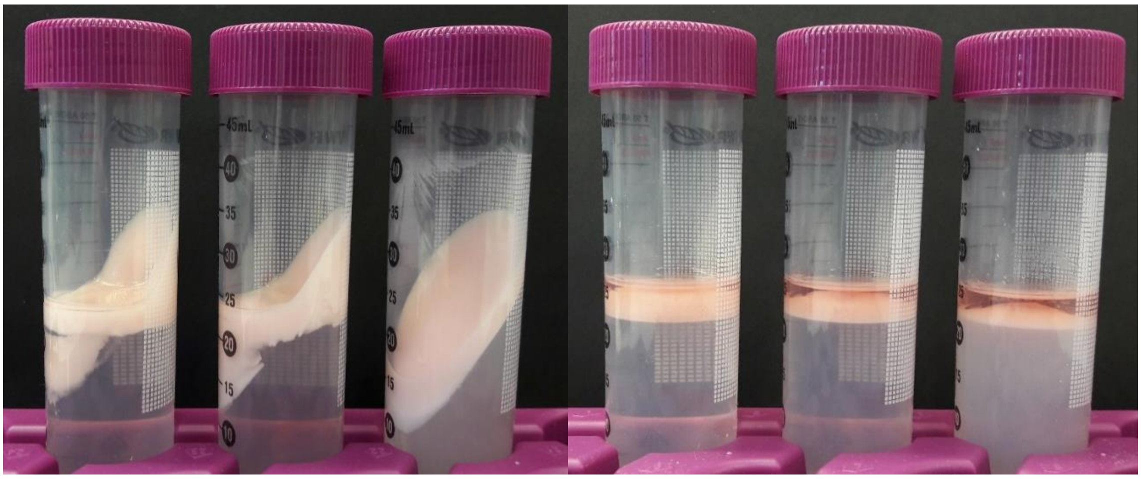

Figure S2: O/w emulsions after the centrifugation (figure on the left) SF-CNF stabilized emulsions, starting from the: nanoparticle dosage of 0.05%, 0.1% and 0.2% (oil amount 10%), and (figure on the right) REF-CNC stabilized emulsions, starting from the left: nanoparticle dosage of 0.05%, 0.1% and 0.2% (oil amount 10%). The red colored liquid is the oil.

"Visual observation of the o/w emulsions after stability testing revealed that the REF-CNCs were insufficient to prevent oil coalescence. Here, the oil formed a separate phase (Supplementary data, Fig. S2). Similar de-stabilization was observed with NON-F-CNFs at all oil and nanoparticle concentrations (results not shown). In contrast, the SF-CNFs prevented coalescence completely at all of the oil dosages studied (i.e., 5, 10, and 20 wt-%—Fig. S2)."
